# Positive Effect of Green Photo-Selective Filter on Graft Union Formation in Tomatoes

**DOI:** 10.3390/plants12193402

**Published:** 2023-09-27

**Authors:** Constanza Carmach, Mónica Castro, Patricia Peñaloza, Leda Guzmán, María José Marchant, Samuel Valdebenito, Iván Kopaitic

**Affiliations:** 1 Laboratorio de Propagación, Escuela de Agronomía, Facultad de Ciencias Agronómicas y de los Alimentos, Pontificia Universidad Católica de Valparaíso, San Francisco S/N, La Palma, Quillota 2260000, Chile; constanza.carmach.p@mail.pucv.cl; 2 Laboratorio de Semillas e Histología Vegetal, Escuela de Agronomía, Facultad de Ciencias Agronómicas y de los Alimentos, Pontificia Universidad Católica de Valparaíso, San Francisco S/N, La Palma, Quillota 2260000, Chile; patricia.penaloza@pucv.cl (P.P.);; 3 Laboratorio de Biomedicina y Biocatálisis, Instituto de Química, Facultad de Ciencias, Pontificia Universidad Católica de Valparaíso, Avenida Universidad 330, Valparaíso 2340000, Chile; leda.guzman@pucv.cl (L.G.);; 4 Laboratorio de Fotometría y Control de Calidad, Escuela de Ingeniería Eléctrica, Facultad de Ingeniería, Pontificia Universidad Católica de Valparaíso, Avenida Brasil 2147, Valparaíso 2340000, Chile

**Keywords:** *Solanum lycopersicum*, grafting, red net, green net, abiotic stress, oxidative stress, acclimation

## Abstract

This study investigated the effects of green and red photo-selective filters (shade nets) on the process of graft union formation (healing and acclimation) in grafted tomato plants. The research evaluated oxidative stress, physiological characteristics, and anatomical development of graft unions. Plants were subjected to green-netting, red-netting, and no-netting treatments for 28 days, starting 4 days after grafting. Markers of oxidative stress, including reactive oxygen species (ROS), superoxide dismutase (SOD), peroxidase (POD), and malondialdehyde (MDA), as well as protein concentration of SOD/POD enzyme-enriched extracts, were quantified. The anatomical development of the graft unions was examined using microscopy. The results demonstrated that the red and green photo-selective filters increased ROS production by 5% and 4% after 3 days of exposure, by 58% and 14% after 7 days, and by 30% and 13% after 14 days in comparison to the control treatment. The increase in ROS activates the defense mechanism, enhancing the activity of SOD and POD enzymes. In terms of anatomy, the green netting resulted in enhanced cell proliferation and early differentiation of vascular tissue cells. Notably, at the 28-day mark, when the plants were ready for transplanting, the green-net treatment showed a reduction in lipid peroxidation damage and increases of 20% and 54% in dry weight compared with the control and red-net treatments, respectively. Finally, our results suggest that the use of a green photo-selective filter has a positive effect on oxidative stress, anatomical development, and overall growth of grafted tomato plants during the process of graft union formation.

## 1. Introduction

Tomato (*Solanum lycopersicum* L.) holds the distinction of being the most cultivated vegetable worldwide [1], but its production faces considerable challenges due to abiotic and biotic stresses. To address these challenges, grafting has emerged as a rapid and efficient technique that improves adaptability and confers resilience to a range of stressors [2]. Grafting in tomato cultivation has gained considerable popularity in various countries, including China, the United States, Italy, Spain, and others [3]. While the primary goal of tomato grafting onto suitable rootstocks is to enhance resistance to soil-borne pathogens, the practice has evolved to include broader goals, such as improving root nutrient uptake, promoting growth, increasing yield, improving fruit quality, and enhancing tolerance to both biotic and abiotic stresses [4,5,6,7,8,9]. 

Grafting is a physical manipulation method whereby two plants are joined by their stems, with one providing the aerial part (the cultivar) and the other providing the rootstock [10]. Following this union, the newly formed plant undergoes two critical processes: the healing of the graft union and the acclimation of the grafted plant [11]. Several biochemical, physiological, and cellular processes take place during the formation of the graft union and collectively determine the timeline and outcome of the procedure. Grafting causes mechanical damage to the plant that sets off responses like the effects of oxidative and osmotic stress. The plant’s physiological defenses to counteract the damage include inhibition of cell elongation; stomatal closure; and reductions in transpiration, stomatal conductance, and CO_2_ assimilation. Photoinhibition and/or photooxidation may also occur [12,13]. 

Furthermore, there is an accumulation of reactive oxygen species (ROS), including superoxide (O_2_**^.^**^−^) and hydrogen peroxide (H_2_O_2_). These ROS have the potential to cause oxidative damage unless counteracted by antioxidant enzymes, namely, catalase (CAT), superoxide dismutase (SOD), and peroxidase (POD) [14]. The SOD enzyme serves as the primary line of defense against ROS by converting singlet oxygen (^1^O_2_ ) to hydrogen peroxide (H_2_O_2_). The POD enzyme plays a complementary role by decomposing hydrogen peroxide to water (H_2_O), like the CAT enzyme [15]. The POD enzyme has an important role in the development of grafted plants. This was highlighted by Fernandez et al. [16], who observed a significant surge in POD activity at the graft site one day after grafting. The increased activity is likely due to the involvement of the POD enzyme in lignin synthesis in response to wounding, as well as its role in the differentiation of new vascular strands [17]. 

Also, the use of green and red phospho-selective filters in grafted plants results in higher ROS accumulation and subsequently lower accumulation of MDA due to the interaction among light quality, photosynthesis, and the antioxidant defense mechanisms of plants [18]. It is possible that the use of green and red phospho-selective filters can alter the balance of the absorbed light energy and affect ROS production during photosynthesis. The higher ROS levels, especially when exposed to red light, trigger antioxidant defense mechanisms, which ultimately reduce the accumulation of MDA by preventing excessive lipid peroxidation. This response reflects the ability of plants to adapt to changes in light quality and manage oxidative stress with their antioxidant regulatory systems [18,19]. Additionally, soluble proteins within plants function as both osmotic regulators and reservoirs for a variety of plant enzymes, playing a pivotal role in governing the growth and metabolism of plant cells [19]. However, during the process of graft union formation, plants begin to accumulate soluble sugars and amino acids, including proline, to protect themselves against potential cellular membrane collapse and the breakdown of cellular compartments [20]. This process also leads to the accumulation of malondialdehyde (MDA), the primary product of lipid peroxidation. As a result of oxidative stress, the accumulation of MDA induces the denaturation of proteins and nucleic acids, as well as the degradation of structural components within cells, affecting the permeability of cell membranes [21].

Tissue adhesion begins with the accumulation of polysaccharides, such as cellulose and pectin, that coat the cell walls [22]. Simultaneously, callus-like masses of undifferentiated cells (known as callus tissue) proliferate adjacent to the existing vascular tissue [23]. Achieving homeostasis allows the plant to rebuild tissues and forge a union between the two components of the graft. A robust vascular connection between the scion and the rootstock determines the physiological functionality of the plant, influencing vital processes such as water and nutrient uptake and translocation, which in turn regulate nutrition, organ growth, photosynthesis, and transpiration [24].

Ensuring optimal environmental conditions during graft union formation is critical to the survival and quality of grafted plants. While comprehensive literature exists on factors such as temperature, relative humidity, and light-intensity conditions in tomato cultivation [15,25,26,27], there is still a lack of information on the effect of light quality on graft healing and acclimation. Light quality refers to wavelength or color, whereas light intensity refers to photon flux density or irradiance. The combined effect of wavelength and light intensity produces a spectrum of incident radiation in the plant environment that affects its growth and development [28].

The spectrum of incident radiation can be modified using LED lighting, supplemental light sources, and photo-selective filters such as polypropylene screens and colored netting. These modifications have the potential to induce a wide range of morphogenetic responses in plants such as stem elongation, plant height, and promotion of flowering, among others [29]. Shading screens (typically white or black) have been used to protect plants from direct solar radiation, thereby reducing temperature, increasing ventilation, and raising relative humidity [30]. In addition, it has been observed that colored netting, acting as a photo-selective filter, alters the light spectrum and thus drives light-mediated physiological responses in plants, including photosynthesis and photomorphogenesis [30].

Previous studies have investigated the effects of different wavelengths within the visible spectrum on plant growth, revealing morpho-physiological, biochemical, and anatomical changes [31,32,33,34,35,36,37,38]. However, current knowledge regarding the influence of colored shade nets during the critical phase of graft union formation is limited. The aim of this study was to fill this gap by performing a comprehensive assessment of metabolic effects on grafted tomato plants exposed to green and red photo-selective filters (shade nets) during the healing and acclimation phases. The analysis included the quantification of oxidative stress, with the purpose of providing detailed insights into the anatomical development of the graft union.

## 2. Results

### 2.1. Oxidative Stress

#### 2.1.1. Determination of Reactive Oxygen Species

**Figure 1:**
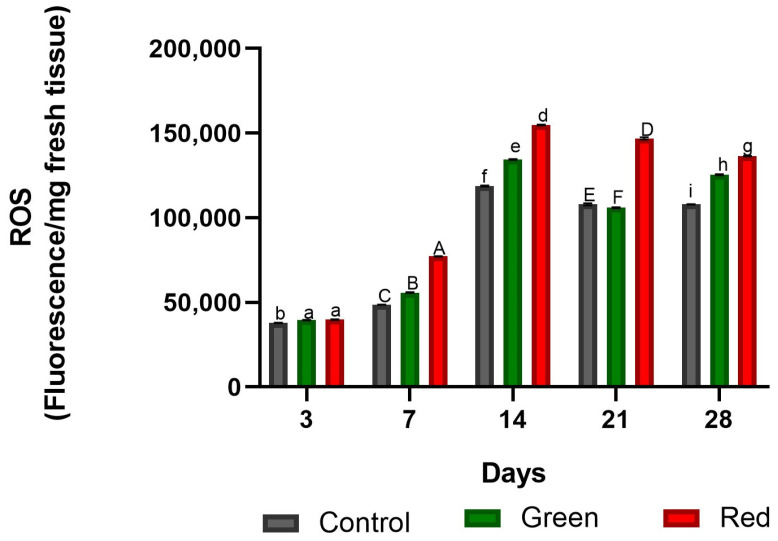
Fluorescence of reactive oxygen species (ROS) at the graft union site in tomato plants subjected to lighting with photo-selective green filter and red filter, and unfiltered control plants. Measurements were made after 3, 7, 14, 14, 21, and 28 days of exposure. Letters (ab, ABC, def, DEF, ghi) indicate statistical differences for each date analyzed with ANOVA and Tukey’s test (*p* < 0.05).

Four days after grafting, photo-selective filters were placed over the plants for a period of twenty-eight days. After three days of exposure, ROS levels in the incision zone showed an increase in both the red-net (5%) and green-net (4%) treatments compared with the control (Figure 1). After seven days of exposure, ROS levels increased by 58% under the red nets and by 14% under the green nets compared with the control (Figure 1). The peak of ROS content in all treatments was reached after 14 days of exposure, with red and green nets showing higher levels compared with the control (30% and 13%, respectively). While ROS production was stabilized after apical incision, the red-net treatment continued to produce higher levels of ROS relative to both the green-net and control treatments.

#### 2.1.2. Superoxide Dismutase Enzyme Activity

**Figure 2:**
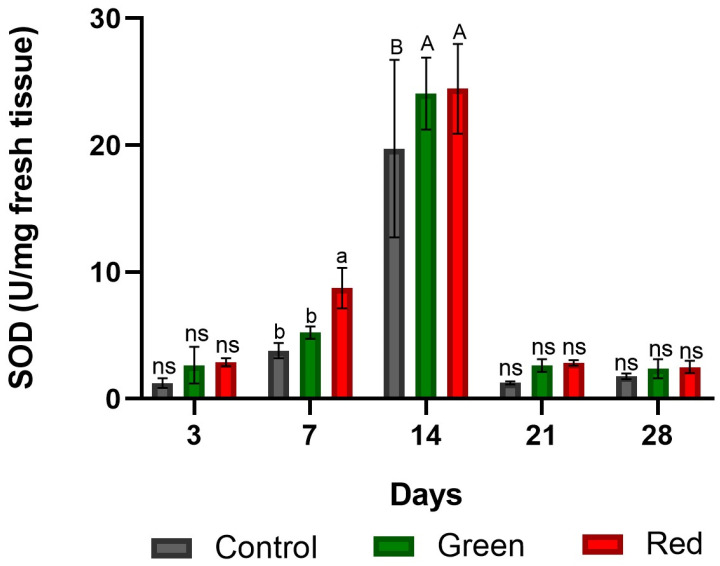
Activity of the enzyme superoxide dismutase at the graft union site in tomato plants subjected to lighting with photo-selective green filter and red filter, and unfiltered control plants. Measurements were made after 3, 7, 14, 21, and 28 days of exposure. Letters (ab, AB) indicate statistical differences for each date analyzed with ANOVA and Tukey’s test (*p* < 0.05). ns: not significant. Bars indicate standard errors.

After 3 days of exposure, no significant differences were observed among the treatments. After 7 days, the red-net treatment exhibited a 129% increase in SOD enzyme activity compared with the control and a 67% increase compared with the green-net treatment. After 14 days of exposure, SOD enzyme activity peaked in all the treatments, with no significant differences between the red- and green-net treatments, but it showed a marked difference compared with the control. After 21 days of exposure, SOD enzyme activity decreased significantly and then stabilized by day 28, with no significant differences observed among the treatments (Figure 2).

#### 2.1.3. Peroxidase Enzyme Activity

**Figure 3:**
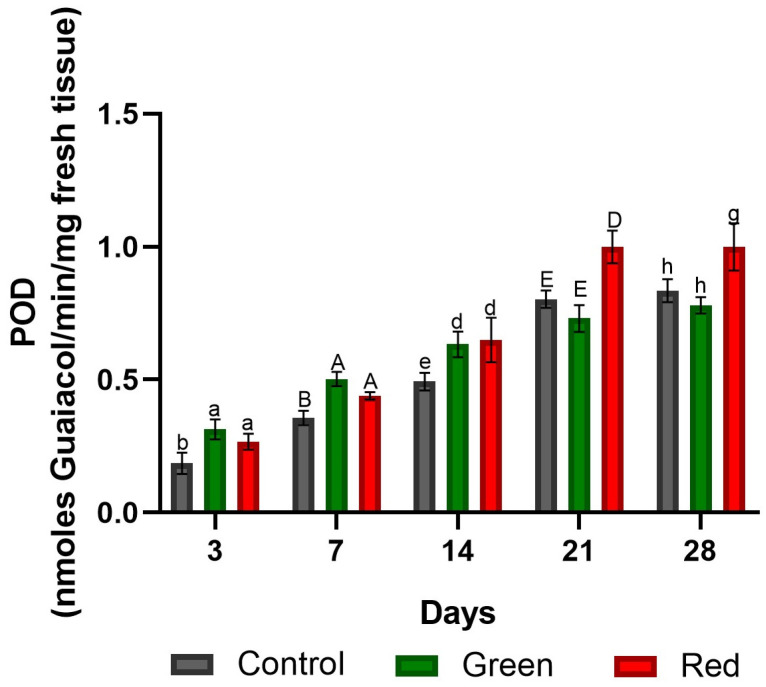
Peroxidase enzyme activity at the graft union site in tomato plants subjected to lighting with photo-selective green filter and red filter, and unfiltered control plants. Measurements were made after 3, 7, 14, 14, 21, and 28 days of exposure. Letters (ab, AB, de, DE, gh) indicate statistical differences for each date analyzed with ANOVA and Tukey’s test (*p* < 0.05). Bars indicate standard errors.

After 3, 7, and 14 days of exposure, higher POD enzyme activity was observed in both the red- and green-net treatments compared with the control, with no significant differences between the two treatments. However, after 21 and 28 days of exposure, the red-net treatment exhibited higher POD enzyme activity than both the control and the green-net treatments (Figure 3).

#### 2.1.4. Determination of Malondialdehyde

**Figure 4:**
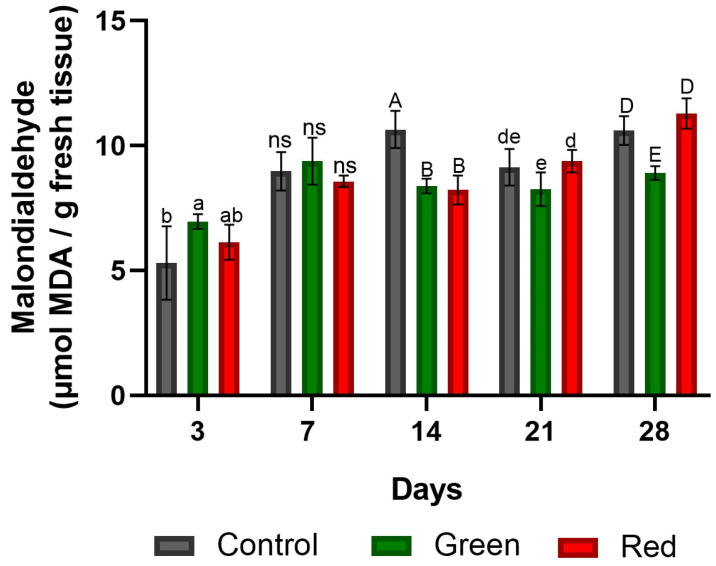
MDA content generated by lipid peroxidation at the grafting site in tomato plants subjected to lighting with photo-selective green filter and red filter, and unfiltered control plants. Measurements were made after 3, 7, 14, 14, 21, and 28 days of exposure. Letters (ab, AB, de, DE) indicate statistical differences for each date analyzed with ANOVA and Tukey’s test (*p* < 0.05). ns: not significant. Bars indicate standard errors.

Tomato plants subjected to the green- and red-net treatments exhibited elevated lipid damage levels (31% and 15%, respectively) after 3 days of exposure compared with the control. However, this trend reversed over time, and by the 14th day of treatment, the control showed 27% and 29% higher MDA content than the green- and red-net treatments, respectively. By the end of the 28-day period, lipid damage in the green-net treatment decreased by 15% in comparison to both the control and the red-net treatments. The results are indicated in the Figure 4.

#### 2.1.5. Protein Concentration of SOD/POD-Enriched Extracts

**Figure 5:**
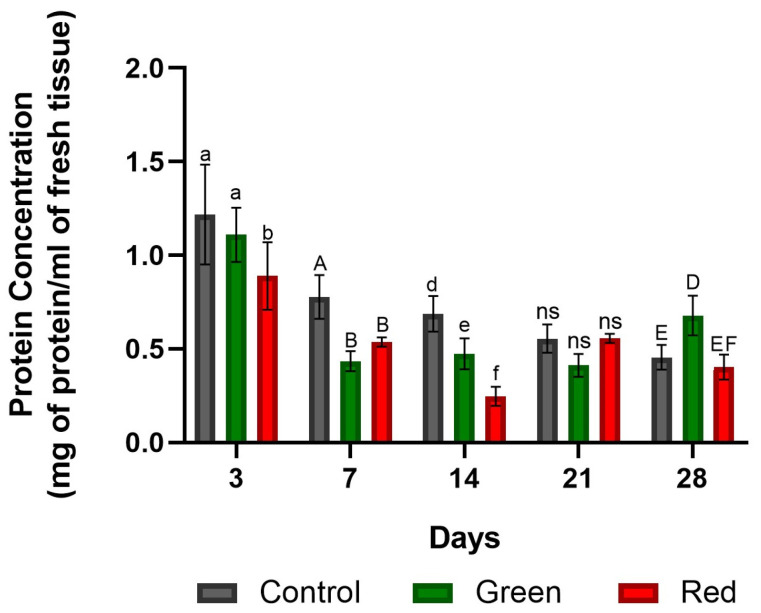
Protein concentration of SOD/POD-enriched extracts at the graft union site in tomato plants subjected to lighting with photo-selective green filter and red filter, and unfiltered control plants. Measurements were made after 3, 7, 14, 14, 21, and 28 days of exposure. Letters (ab, AB, def, DEF) indicate statistical differences for each date analyzed with ANOVA and Tukey’s test (*p* < 0.05). ns: not significant. Bars indicate standard errors.

After 3 days of exposure, no significant differences were observed between the unfiltered control and the green-net treatment. However, both had a higher concentration of protein compared with the red-net treatment. After 7 days of exposure, the control showed increased total protein content, while no significant differences were observed between the green- and red-net treatments (Figure 5). After 14 days of exposure, the control maintained a higher concentration of protein compared with the other treatments. After 21 days of treatment, no significant differences were observed among the treatments. However, by day 28, the green-net treatment showed increased protein content (Figure 5).

### 2.2. Anatomical Development

**Figure 6:**
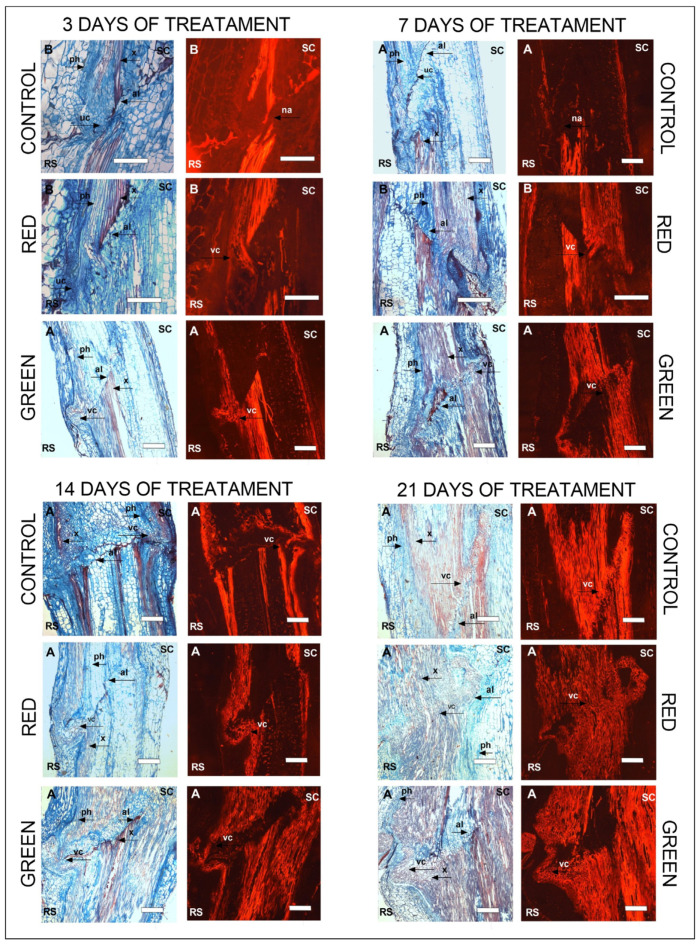
Longitudinal stem sections corresponding to graft union after 3, 7, 14, and 21 days of exposure. Images are shown under bright-field microscopy and are contrasted with fluorescence microscopy to compare the development of the vascular connection. Rootstock (RS), scion (SC), adhesion line (al), no adhesion (na), vascular connection (vc), undifferentiated cells (uc), xylem (x), phloem (ph). White box indicates the scale bar: 250 μm (A) and 200 μm (B).

After 3 days of exposure, significant differences in vascular tissue development were observed, with the green-net treatment showing markedly superior xylem development and union between rootstock and cultivar (Figure 6). After 7 days, both the red- and green-net treatments showed more advanced vascular tissue development in contrast to the control, which had weaker union. However, between days 14 and 21, there was greater xylem fluorescence in the control treatment, although the graft line remained distinguishable. In contrast, the other treatments showed more advanced xylem development and a stronger union over this time, with only a faintly visible graft line.

**Figure 7:**
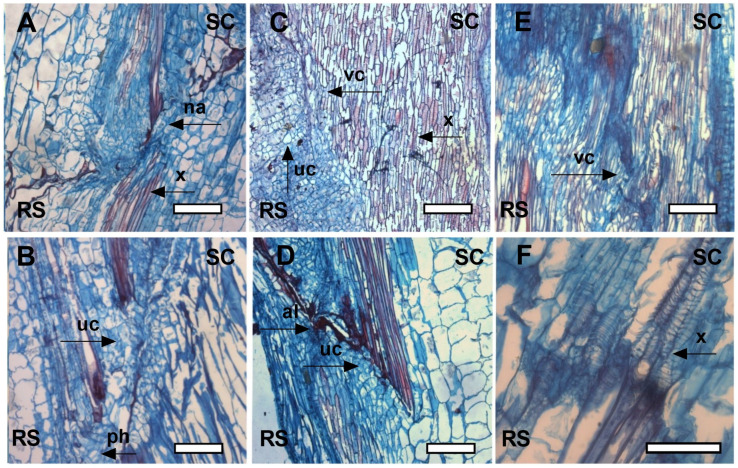
Longitudinal sections of stems in the graft union zone after 3 days of exposure (control (**A**,**B**); red (**C**,**D**), and green (**E**,**F**) photo-selective filters). Rootstock (RS), scion (SC), adhesion line (al), no adhesion (na), vascular connection (vc), undifferentiated cells (uc), xylem (x), phloem (ph). White box indicates the scale bar: 200 μm (**A**–**E**) and 100 μm (**F**).

After 3 days of exposure, cell proliferation was noted near the vascular system in the control treatment (Figure 7A,B), and masses of undifferentiated cells with indistinct nuclear features could be seen under the microscope. In the red-net treatment (Figure 7C,D), the cell masses began to differentiate into xylem vascular tissue, although the cells were still smaller and/or shorter than mature xylem cells. In the green-net treatment (Figure 7E,F), cell masses were identified in a significantly more advanced stage of differentiation compared with the control, similar to the red-net treatment but with slightly more elongated xylem cells, indicating further progression of cell elongation.

### 2.3. Fresh and Dry Plant Weight 

**Figure 8:**
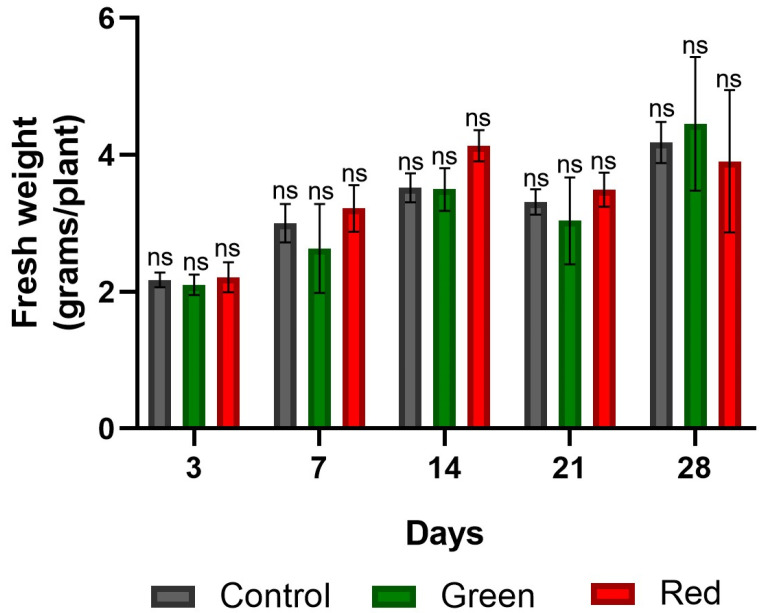
Fresh weight of tomato plants subjected to lighting with photo-selective green filter and red filter, and unfiltered control plants. Measurements were made after 3, 7, 14, 21, and 28 days of exposure. Analyzed with ANOVA and Tukey’s test (*p* < 0.05). ns: not significant. Bars indicate standard error.

**Figure 9:**
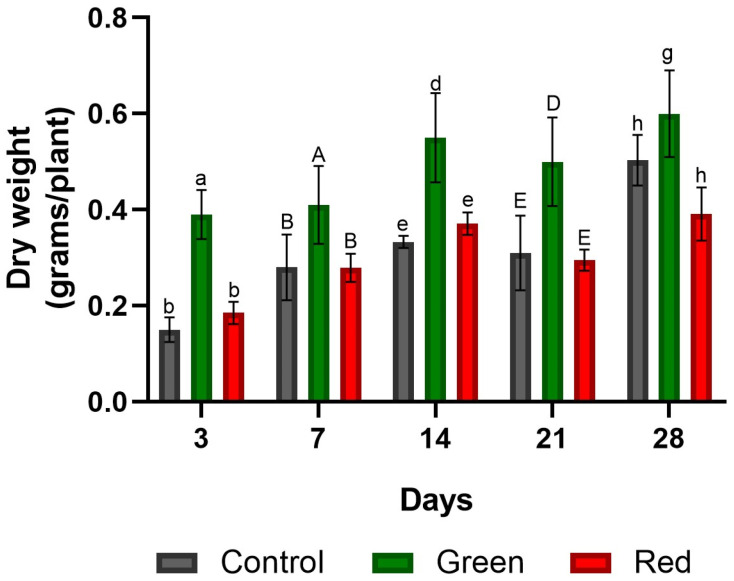
Dry weight of tomato plants subjected to lighting with photo-selective green filter and red filter, and unfiltered control plants. Measurements were taken after 3, 7, 14, 21, and 28 days of exposure. Letters (ab, AB, de, DE, gh) indicate statistical differences for each date analyzed with ANOVA and Tukey’s test (*p* < 0.05). Bars indicate standard error.

**Figure 10:**
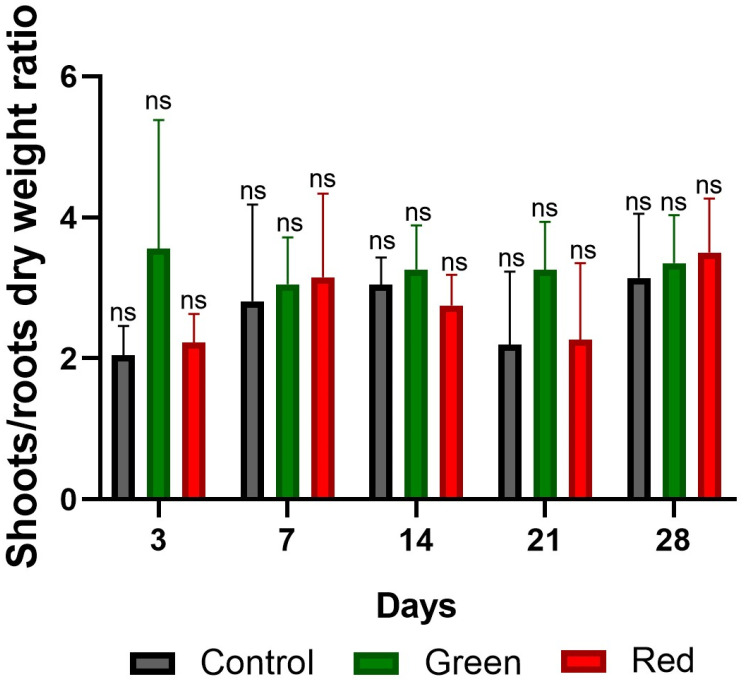
Ratio of dry weight of aerial parts and roots of grafted tomato plants after 3, 7, 14, 21, and 28 days of exposure to photo-selective green filter, red filter, and unfiltered control plants. Analyzed with ANOVA and Tukey’s test (*p* < 0.05). ns: not significant. Bars indicate standard error.

No significant differences in fresh weight were observed among the treatments (Figure 8). However, in terms of dry weight, the green-net treatment consistently outperformed the others in all measurements. After only 3 days of exposure, the green-net treatment showed dry weight increases of 160% and 110% compared with the control and red-net treatments, respectively. Upon reaching the transplanting stage at the 28-day mark, the green-net treatment showed dry weight gains of 20% and 54% over the control and red-net treatments, respectively (Figure 9). Initially, after 3 days of exposure, the use of green netting resulted in greater dry matter accumulation in tomato shoots relative to roots, in contrast to the control and red-net treatments. However, by the 28-day mark, as the plants approached transplant readiness, all treatments had similar shoot-to-root ratios, with shoot growth dominating (Figure 10).

## 3. Discussion

Previous research has shown that plant responses vary depending on the type of incident radiation. These responses can include adverse effects, such as cell membrane damage, reduced photosynthetic rates, and premature aging. Conversely, they can also have beneficial effects, such as accelerated growth and increased stress resistance, achieved by activating antioxidant responses to mitigate the effects of ROS [39]. ROS production has a significant role in various cellular processes, such as cell signaling, and it can be involved in graft union formation in plants. In fact, our study showed that the use of green and red photo-selective filters led to increased ROS production at the graft union site compared with the control. Although an increase in ROS is harmful, fine control of ROS production is a signal to activate plant defense mechanisms, promoting the activation of antioxidant enzymes and other protective responses to prevent infection and promote healing after grafting [17,18,27,40]. Therefore, the increase in ROS is related to the increase in SOD and POD enzymes in the graft union area. 

After 21 days of exposure, a reduction in ROS levels was observed in all treatments, consistently with a marked decrease in SOD activity. This pattern could be attributed to (i) a positive union and anatomic development (advanced xylem development and a stronger union); (ii) the apical incision performed on day 15 of treatment to facilitate the formation of guide shoots. This new incision likely caused damage that required an immediate antioxidant response.

In addition, the antioxidant response on day 7 of exposure resulted in a significant increase in SOD enzyme activity in the red-net treatment, an effect that was replicated on day 14 in the green-net treatment—both significantly different from the control. In essence, both filters appeared to induce analogous antioxidant responses at the graft site.

The effect of red-net treatment is consistent with the findings obtained by Manivannan et al. [41], who observed elevated levels of SOD and other antioxidant enzymes in *Dianthus caryophyllus* explants after 8 weeks of exposure to red LED light (450 nm). The increase correlated with improved explant growth in terms of both length and root count, as well as improved photosynthetic parameters. This favorable outcome may be related to increased levels of endogenous hormones, including cytokinins and gibberellins, which are known to facilitate cell division, differentiation, and elongation.

Conversely, Xu et al. [42] showed that green light enhanced in vitro root growth of *Cunninghamia lanceolata* plants, in part due to increased activity of the antioxidant enzymes SOD and POD. In addition, green light inhibited the accumulation of MDA, a by-product of lipid peroxidation. Our results are consistent with this observation, as both filters similarly increased POD enzyme activity in response to ROS levels. The increase in the POD enzyme could reduce the levels of substrates necessary for lipid peroxidation, resulting in reduced MDA levels compared with the control. Likewise, in vitro cultured *Withania somnifera* L. plants exposed to green light exhibited greater SOD production than those exposed to red light and the white-light control. As indicated in the introduction of this manuscript, low accumulation of MAD might indicate the ability of acclimatized and grafted plants to adapt to photo-selective filters.

Furthermore, the production of POD was more pronounced under the influence of green light compared with the control, whereas no significant difference was observed in the red-light treatment. Both enzymes play a key role in mitigating the effects of ROS generated during plant metabolism. As the first line of defense, the SOD enzyme catalyzes the conversion of superoxide anion radicals to hydrogen peroxide. The POD enzyme, in turn, aids in the oxidative breakdown of hydrogen peroxide into co-substrates, such as phenolics or other antioxidant compounds [14].

Specifically, in the context of grafting, Li et al. [43] incorporated green-light exposure for recently grafted tomato plants, resulting in improved survival rates and increased activity of antioxidant enzymes such as peroxidase. This improvement was evident in an accelerated vascular regeneration process, with the POD enzyme catalyzing lignin synthesis in vascular tissues and tracheary elements. In support of these findings, Fernandez et al. [20] strengthened the argument by demonstrating that the POD enzyme was concentrated around the graft union region, with its increase correlating with xylem lignification.

Our results support this information, as we observed increased proliferation and early differentiation of vascular tissue cells (Figure 6 and Figure 7) under the influence of both red and green netting. In contrast, the control group showed delayed cell differentiation. In particular, the green net induced xylem differentiation as early as day 3 of treatment (equivalent to 7 days after grafting), as shown in Figure 6 and Figure 7, while the green net accelerated tissue union, a phenomenon supported by studies by Fan et al. [44] and Frey et al. [23]. These studies showed the existence of functional connections between specific graft regions, and the xylem and phloem of the rootstock as early as 10 days after grafting. While lignin was usually present in non-union areas, its levels decreased as the graft healed, consistently with the observations by Frey et al. [23]. 

Our results show that the application of the green-net treatment resulted in increased total plant dry weight compared with both the control and red-net treatments (Figure 9). Interestingly, these results differ from those reported by Adil et al. [18], who found higher dry weight under red light than under green light in *Withania somnifera* L. plants. This discrepancy suggests that the involvement of red-light receptors could stimulate tissue formation by enhancing the transport and biosynthesis of growth regulators. In our investigation, the red-net treatment showed no significant differences in dry weight compared with the control. This observation is consistent with the study by Lara et al. [45], who applied a red net to *Spinacia oleracea* L. (spinach) plants and compared them with an unfiltered control.

On the contrary, alternative studies have shown that the incorporation of green light in basil (*Ocimum basilicum* L.) cultivation resulted in higher biomass accumulation in both fresh and dry weight compared with the control under white-light conditions [34]. In addition, Kaiser et al. [31] documented increases in total fresh and dry weight in tomato corresponding to increased green-light intensity. Similarly, Johkan et al. [46] highlighted the ability of green light to promote biomass production in lettuce (*Lactuca sativa*). Specifically, the introduction of green light has been shown to enhance the healing process of grafted seedlings and stimulate root development, thereby increasing root dry weight and overall seedling quality [44]. 

It is worth noting that the evidence of accelerated root growth under green light contradicts our own observations. Specifically, during the first 3 days of exposure, the green-net treatment showed a more pronounced increase in shoot growth compared with root growth. Similarly, at the end of the 28-day treatment, when the plants were ready for transplanting, all treatments showed an increased shoot-to-root ratio. This shift is likely due to the apical cut made to promote the proliferation of guide shoots.

The increase in biomass can also be attributed to the phenomenon that green light is absorbed by leaves deeper in the canopy, as opposed to red wavelengths [47]. In addition, plants use specific mechanisms to adapt to different environments. For example, under conditions of light deficiency, plants increase their photosynthetic pigments, thereby boosting the efficiency of light absorption and optimizing carbon balance [48]. In addition, different wavelengths of light affect sugar metabolism in plant cells, leading to variations in biomass production [49].

Furthermore, Bian et al. [32] demonstrated that green light could enhance plant resilience to both biotic and abiotic stressors. This is achieved by inducing differential gene expression, minimizing stomatal aperture, increasing water-use efficiency, and maintaining elevated photosynthetic capacity under short-term drought stress. Similarly, red netting has been used to counteract salt stress in pepper (*Capsicum annuum* L.) by increasing cytokinin and abscisic acid concentrations and enhancing salicylic acid levels, resulting in shoot growth recovery [50]. 

Yousef et al. [38] showed that a 7:3 mixture of red and blue light significantly increased the expression of auxin-related genes in grafted tomato plants, accelerating cell division and proliferation. However, red light alone hinders the differentiation of vascular tissues and may induce callus formation on both sides, leading to graft failure or an ineffective arrangement [38]. As discussed above, total protein content serves as a reservoir for the synthesis of growth-promoting enzymes and hormones. Therefore, it represents the active state of the cell: the higher the total protein content, the higher the gene expression and transcription to execute and process the light signal perceived by the cell [14].

In addition to the above described results, the Table 1 shows the effects of different photo-selective filters on plants such as tomato, spinach, peppers, etc., along with our results.

## 4. Materials and Methods

### 4.1. Growth Conditions

Tomato (*Solanum lycopersicum* L.) plants were produced at the Agronueve S.A. nursery in Quillota, Valparaíso, Chile (−32.8981513, −71.2591605). The propagation process started with the sowing of the variety “Alamina” and the rootstock “Suzuka”, both from Rijk Zwaan, in a mixture of peat and perlite substrate in a 70:30 (%*v*/*v*) ratio in expanded polystyrene trays of 135 cells, in a double-roofed polyethylene greenhouse. Thirty days after planting, the plants reached a stem thickness of about 3 mm and were disinfected two days before grafting with Previcur^®^ (Bayer, Leverkusen, Germany) 1.5 cc/L (fungicide: Propamocarb and Fosetyl) and Nacillus^®^ (Microbial Discovery Group, Oak Creek, WI, USA) 3 g/L (biological bactericide: *Bacillus* spp. and *Brevibacillus brevis*). Inside the grafting chamber, the stems of the plants were cut at an angle of 60°, 2 cm above the cotyledons for the scion and below the cotyledons for the rootstock. Strepto Plus^®^ (IndiaMART, Noida, India) (bactericide: streptomycin sesquisulfate and oxytetracycline hydrochloride) was applied at a concentration of 1 g/L, and both parts were secured with a silicone clip using a simple splicing technique. Throughout the grafting process, the plants were fertilized with Ultrasol^®^ (SQM Specialty Plant Nutrition, Santiago, Chile) multi-purpose fertilizer (18-18-18) at rates of 0.2 to 0.7 g/L. Fungicides, bactericides, and insecticides such as Proplant^®^ (IndiaMART, Noida, India) (propamocarb-HCl), Agrygent^®^ (Summit Agro, Durham, NC, USA) (gentamicin sulfate + oxytetracycline hydrochloride), and Proclaim^®^ (Syngenta, Basel, Switzerland) (emamectin benzoate) were applied intermittently. A total of 270 plants per treatment were used as destructive samples, placed in an incubator chamber, and exposed to the treatments for a period of 28 days (Table 2). These treatments included the use of a green photo-selective filter, a red photo-selective filter, and a control with no photo-selective filter. After 49 days of growth, the apical tissue was excised to stimulate the growth of two lateral guide shoots.

**Figure 11:**
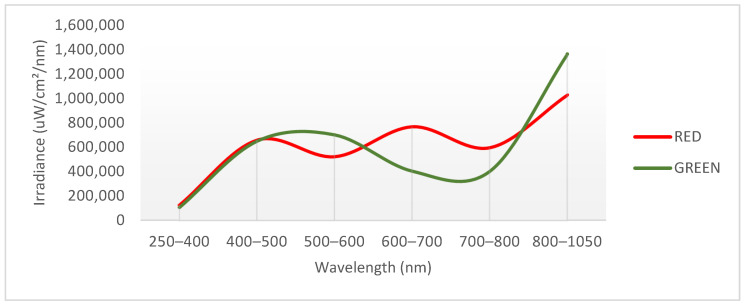
Measurement of the quality of the light irradiated under the red and green nets carried out in the photometry laboratory, Faculty of Electrical Engineering, PUCV.

High-density polyethylene (HDPE) screens with 50% opacity were placed 2 m above the plants, maintaining a distance to avoid overlap and interference. To characterize the photo-selective filters, irradiance was measured in the laboratory using a spectrophotometer with an incandescent lamp. This procedure provided light-intensity data at various wavelengths. These raw values were then correlated with sunlight irradiance data specific to the Quillota region to produce a prototype radiation spectrum generated by the filters (Figure 11).

### 4.2. Reactive Oxygen Species Activity Measurement

To evaluate ROS, 200 mg of tissue corresponding to the graft union site (approximately 1 cm above and 1 cm below the incision) was extracted, ground in a mortar, and homogenized in 1 mL of 10 mM Tris-HCl, pH 7.2 (extraction buffer). Samples were centrifuged at 12,000× *g* for 20 min at 4 °C. The supernatant was then collected and diluted in extraction buffer to measure its fluorescence with and without 1 mM DMCA probe. Fluorescence was related to the level of proteins present in the tissue to obtain the ROS content [51].

### 4.3. Antioxidant Enzymes

#### 4.3.1. Extract

To evaluate the effect of activity of antioxidant enzymes on the ROS generation, samples were extracted by weighing 200 mg of tissue corresponding to the graft union site (approximately 1 cm above and 1 cm below the incision), crushing it in a mortar with a pestle, and homogenizing it in 1.2 mL of 0.2 M phosphate buffer (pH 7.8 with 0.1 mM EDTA). Samples were centrifuged at 15,000× *g* for 20 min at 4 °C. The supernatant was collected, and the pellet was resuspended in 0.8 mL of the same buffer and centrifuged at 15,000× *g* for 15 min. Finally, both supernatants were combined to determine the activity of SOD and POD enzymes [51]. Protein concentration of SOD/POD enzyme-enriched extracts was determined using the bicinchoninic acid (BCA) method.

#### 4.3.2. Superoxide Dismutase

Total SOD activity was assayed using NBT, a yellow compound that is reduced to blue monoformazan by the superoxide radical and is thus quantified as a function of the competitive inhibition of NBT reduction by the superoxide radical. The 2 mL assay reaction mixture contained 50 mM phosphate buffer (pH 7.8) with 2 mM EDTA, 9.9 mM L-methionine, 55 μM NBT, 0.025% Triton-X100, 40 μL of sample extract, and finally 20 μL of 1 mM riboflavin to initiate the reaction. The samples were illuminated with a 15 W fluorescent light at 12 cm for 10 min while oscillating on an orbital shaker [51]. The blank was kept in the dark. When the reaction was stopped, the absorbance at 560 nm was measured and extrapolated from a standard curve obtained with pure SOD [52].

#### 4.3.3. Peroxidase

The assay mixture (1 mL) contained 50 mM potassium phosphate buffer (pH 7.0), 0.5 mM guaiacol, 0.5 mM H_2_O_2_, and 10 μL of sample extract. H_2_O_2_ was added at the end to initiate the reaction, and the absorbance was recorded for 3 min. The increase in absorbance at 470 nm due to guaiacol oxidation was measured. The activity was estimated using the extinction coefficient of 26.6 mM/cm [53].

### 4.4. MDA

To evaluate the effect of ROS on tissue damage, lipid peroxidation was measured using the thiobarbituric acid (TBA) test, which determines malondialdehyde (MDA) as a product of lipid peroxidation. The assay was performed as described by Velikova et al. [54]. In brief, 500 mg of plant material was collected from the graft union site and homogenized in 5 mL of a 0.1% (*w*/*v*) solution of TCA. The homogenate was then centrifuged at 10,000× *g* for 20 min, and 0.5 mL of the resulting supernatant was mixed with 1 mL of 0.5% (*w*/*v*) TBA in 20% TCA in a separate tube. The mixture was then heated in boiling water for 30 min, after which the reaction was stopped by placing the reaction tubes in an ice bath. The samples were subsequently centrifuged at 10,000× *g* for 5 min, and the absorbance of the supernatant was measured at 532 nm using a spectrophotometer. The non-specific absorbance at 600 nm was subtracted. The quantity of MDA-TBA complex was determined based on the extinction coefficient of 155 mM/cm.

### 4.5. Anatomical Development

Segments were excised from the graft union 3, 7, 14, and 21 days after transplantation (DDI) for the analysis of tissue differentiation and the observation of vascular and supportive tissues. The protocol, adapted from Chamberlain [55], Johansen [56], D’Ambrogio de Argüeso [57], and Singh and Mathur [58], involved the following steps: tissue fixation in F.A.A. solution, dehydration using ascending alcohol concentrations, clearing with xylene, and impregnation with Paraplast^®^ (Leica Biosystems, Heidelberger, Germany) kerosene. Histologic sections were prepared with thickness of 10 μm, taken 1 cm above and 1 cm below the graft site, using a Thermo Scientific™ (Waltham, MA, USA) HM 325 rotary microtome. The sections were then stained with safranin-fast green following the protocol by D’Ambrogio de Argüeso [57]. The specimens were examined using an Olympus (Tokyo, Japan) CX31 epifluorescence microscope at a total magnification of 400× Finally, images were captured using a QImaging Mi-croPublisher 3.3 RTV camera (Teledyne QImaging, Surrey, BC, Canada) and analyzed with QImaging QCapture Pro 5.1 software.

### 4.6. Fresh and Dry Weight

The plants were separated at the point of grafting, and both segments (shoots and roots) were dried in an oven at a constant temperature of 75 °C for 24 h. The ratio of shoots to roots was then calculated according to Temperini et al. [59] by dividing the dry weight of the shoots by the dry weight of the roots (in grams).

### 4.7. Statistical Analysis

The experiment used a completely randomized design with a total of 270 plants per treatment. The experimental unit (*n*) consisted of 6 grafted plants for each measurement. Normality and log-normality of the data were assessed using the Shapiro–Wilk test. Two-way analysis of variance (2-way ANOVA) was performed with a significance level of *p* < 0.0001, followed by Tukey’s multiple comparison test with a 95% confidence interval. A difference was considered statistically significant when *p* < 0.05. All statistical analyses were performed using GraphPad Prism 8 software (GraphPad Software, San Diego, CA, USA).

## 5. Conclusions

The results of this study demonstrated that red and green photo-selective filters increased ROS production by 5% and 4% after 3 days of exposure, by 58% and 14% after 7 days, and by 30% and 13% after 14 days in comparison to the control treatment. The increase in ROS caused elevated production of SOD and POD enzymes during the process of graft union formation. In terms of anatomy, the green netting resulted in enhanced cell proliferation and early differentiation of vascular tissue cells. Notably, at the 28-day mark, when the plants were ready for transplanting, the green-net treatment showed a reduction in lipid peroxidation damage and increases of 20% and 54% in dry weight compared with the control and red-net treatments, respectively. These results suggest that the use of a green photo-selective filter can positively influence oxidative stress, anatomical development, and overall growth of grafted tomato plants during the process of graft union formation.

## Figures and Tables

**Table 1:** The effects of different photo-selective filters on plants such as tomato and other plants.

Species	Treatment	Effect	Reference
*Solanum lycopersicum* L. cv. Komeett	Green light	Increases in stem length, and fresh and dry weight. Leaf size is reduced	[31]
*Solanum lycopersicum* L. cv. Ailsa Craig	Green light	Under water stress conditions, lipid peroxidation (MDA) decreased, and photosynthetic capacity was maintained.	[32]
*Solanum lycopersicum* cv. Ingar F1	Red and green light	Green light increased the efficiency of light use in young plants, resulted in stem elongation, and decreased biomass compared with red light, which also increased the photosynthetic rate.	[33]
*Ocimum basilicum* L.	Green light	Biomass, number of leaves, stem length, and leaf area increased.	[34]
*Ocimum* sp.	Green net	Fresh and dry weight increased, and so did the number of shoots/plant.	[35]
*Spinacia oleracea* L.	Red net	Fresh weight, total phenolic content, and antioxidant capacity increased.	[45]
*Capsicum annuum* L.	Red net	Increased water-use efficiency, phytohormone expression, and plant height.	[50]
*Solanum lycopersicum* *Mill*. var. “Sida”	Red net	Increases in biomass, shoot/root ratio growth, height, and leaf area.	[37]
*Capsicum annuum* L.	Red and green net	Both increased leaf area and commercial yield.	[36]
*Solanum lycopersicum* L. cv. Gangmu No. 1	Red and blue light	Promoted rapid cell proliferation	[38]
*Cunninghamia lanceolata*	Green light Tissue culture	Enhanced root growth in tissue culture. Increased the production of antioxidant enzymes, and decreased lipid peroxidation.	[42]
*Solanum lycopersicum* L. cv. Beaded Curtain and Rootstock No. 1	Green light	Increased the production of antioxidant enzymes and improved vascular regeneration.	[43]
*Withania somnifera* L.	Red light Tissue culture	Increased callus formation in tissue culture, biomass accumulation, and antioxidant activity.	[19]

**Table 2:** Chronological stages of the production process for grafted plants and the duration of exposure. h: hour, RH: relative humidity.

Stage or Process	Duration of Stage	Duration of Exposure	Total Age of Plant	Environmental Conditions
Sowing and growth	30 days	0 days	30 days	92.8% RH and 16.2 °C
Grafting chamber	3 h	0 days	30 days	96.8% RH and 17.3 °C
Incubation chamber	4 days	0 days	34 days	98.8% RH and 24.5 °C
Curing chamber 1	3 days	3 days	37 days	96.8% RH, 17.1 °C heating with furnace, and the opening/closing of curtains during the day/night
Curing chamber 2	4 days	7 days	41 days	92.4% RH, 16.4 °C, and the opening/closing of curtains during the day/night
Acclimation	7 days	14 days	48 days	92.9% RH and 15.1 °C
Apical incision	2 h	15 days	49 days	92.9% RH and 15.1 °C
Shoot growth 1	7 days	21 days	55 days	92.7% RH and 14.9 °C
Shoot growth 2	7 days	28 days	62 days	92.6% RH and 14.9 °C
**Ready plant**			**62 days**	

## Data Availability

Data sets generated for this study are available upon request from the corresponding author.
